# Higher prevalence of permanent congenital hypothyroidism in the Southwest of Iran mostly caused by dyshormonogenesis: a five-year follow-up study

**DOI:** 10.20945/2359-3997000000085

**Published:** 2018-10-01

**Authors:** Majid Aminzadeh

**Affiliations:** 1 Ahvaz Jundishapur University of Medical Sciences Ahvaz Jundishapur University of Medical Sciences School of Medicine Pediatric Department Ahvaz Iran Division of Pediatric Endocrinology and Metabolism, Pediatric Department, School of Medicine, Ahvaz Jundishapur University of Medical Sciences, Ahvaz, Iran

**Keywords:** Congenital hypothyroidism, dyshormonogenesis, screening, Tc-99m thyroid scintigraphy, thyroid dysgenesis

## Abstract

**Objective:**

The incidence of congenital hypothyroidism (CH) varies globally. This 5-year study aimed to determine the prevalence of permanent CH in the southwest of Iran.

**Materials and methods:**

Between January 2007 and December 2009, all newborns in Ahvaz, the biggest city in the southwest of Iran, were screened for CH using a heel-prick sample for thyrotropin (TSH) levels. Subjects with TSH ≥ 5 mU/L were evaluated for T4-TSH. Infants with T4 < 6.5 µg/dL, TSH > 10 mU/L, and normal T4 but persistent (> 60 days) high TSH were considered to have CH. After the third birthday, treatment was discontinued, and T4-TSH was reevaluated; subjects with TSH ≥ 10 mU/L were investigated using thyroid Tc99 scintigraphy (TS). Based on TS, they were classified as normal, dysgenetic, or athyretic (agenesis).

**Results:**

Screening was performed for 86,567 neonates, and 194 were confirmed to have CH (100 males; F/M = 0.94; overall incidence 1:446). After the third birthday, reevaluation was performed in all (except 18 that were not accessible). From 176 patients, 81 (46%) were diagnosed with permanent CH, and 95 were discharged as transient. Considering the same percentage in the lost cases, the prevalence of permanent CH was found to be 1:970. TS performed for 53 of the permanent subjects found agenesis/dysgenesis in 25 (F:M = 15:10) and a normal result in 28 (F:M = 11:17), indicating dyshormonogenesis as the cause in more than 50% of subjects.

**Conclusions:**

The incidence of CH in this area was found to be higher than that in other countries but less than the incidence rate reported in central Iran. The large number of transient cases of CH suggests environmental or maternal causes for the incidence rather than a genetic basis.

## INTRODUCTION

Screening for congenital hypothyroidism (CH), one of the most common causes of preventable mental retardation, has been performed in developing countries since the 1960s, but it has been routinely conducted in Iran only since the year 2000. The prevalence of CH has been reported to be 1/1,000 to 1/370 in Iran (1,2), which is significantly higher than that of the American and European countries (1/4,000) (3,4). The positive cost–benefit ratio of screening for CH ranges from 3.6:1 in developed countries (5) to 15:1 in developing countries such as Iran (6). Theoretically, dysgenesis, dyshormonogenesis, and iodine deficiency are the most common causes of CH. Prenatal (iodinated disinfectants) and postnatal iodine exposure (milk iodine content) have also been implicated as etiologic factors (7,8). Varying incidence of CH has been reported in different areas of Iran, with no data from the southwest regions of the country, which is different from central and northern Iran in terms of weather, altitude, and gene pool. Another difference between various areas of Iran is that the southwest regions of the country have a higher rate of consanguineous marriages than the other regions. The latest reported CH incidence of 1/370 neonates in central Iran with a large number of transients (40%) (9) was significantly higher than the incidence rate previously reported. Accordingly, we aimed to determine the prevalence of CH in the southwest of Iran using a more definite diagnostic algorithm in a large enough population and for a longer duration. The classic approach used herein may provide guidelines for diagnosing recalled (or referred) infants by a standard and safe algorithm (fully described in Materials and Methods and Table 1).

**Table 1:** Study protocol for decision-making in the screening program of CH

T4 (g/dL)	TSH^a^ (mU/L)	Decision	Follow-up
≥ 8.5	< 5.5	Discharge	None
≤ 6.49	≥ 10	Treatment	As a CH^b^ case
6.5–9.9	≥ 10	Suspected CH	TFT 2 wks later
6.5–9.9	5–9.9	Suspected CH	TFT 4 wks later
≥ 10	≥ 5	Suspected CH	TFT 4 wks later
Each level	> 6.0 after 60^th^ day (10)	Treatment	As a CH case

a Thyroid stimulating hormone (normal for 2-20 wks = 0.5–5.5);

b congenital hypothyroidism.

## MATERIALS AND METHODS

**Figure 1:**
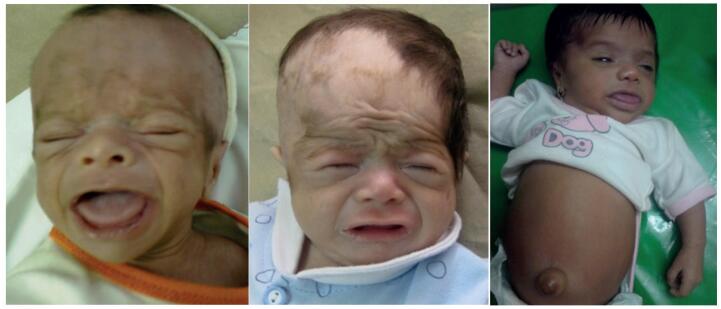
Three cases of congenital primary hypothyroidism were missed because of parental negligence, referred at 3 months with developmental delay.

**Figure 2:**
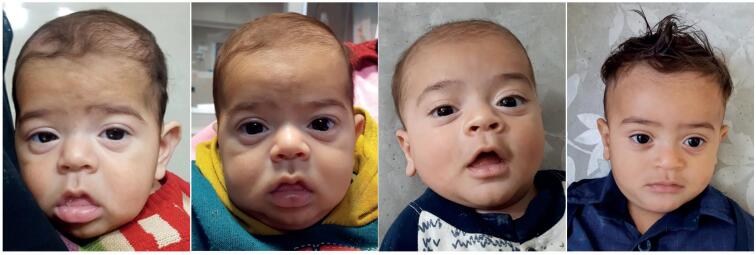
A 6-month-old infant referred with poor growth diagnosed as congenital central hypothyroidism. He was missed because of screening with TSH. Serial photos (left to right: at the time of diagnosis, 1 week, 4 weeks, and 6 months after treatment) clearly show the facial changes as a reliable marker of clinical response over time.

During the 3-year period from January 2007 to December 2009, all infants born in Ahvaz, the biggest city in the southwest of Iran, were enrolled (Table 2). This study was approved by the Ethical Committee of Ahvaz Jundishapur University of Medical Sciences. All neonates were screened before getting their identification card to ensure 100% compliance (however, some infants who did not pursue this policy were missed; Figure 1). Individuals who were born in Ahvaz but whose parents lived in other cities were excluded but were followed up on and treated if required. In all healthy and term babies, blood was obtained from a heel prick between the 3^rd^ and 5^th^ day of life and was collected on a filter paper for thyroid stimulating hormone (TSH) assessment using a kit (Padtan Elm Incorporation, Tehran, Iran) and an ELISA reader (Stat Fax 2100; Awareness Technologies, USA) by the same four staff members during the study period. For preterm infants and those who required postnatal hospitalization, testing was done after discharge but before the 30^th^ day of life. The subjects were notified before the 10^th^ day if the TSH level was > 5 mU/L and were referred for suspected CH. Screening in this study was based on TSH measurement, so it may not have detected infants with delayed TSH elevation or with central or hypopituitary hypothyroidism (Figure 2). Infants with TSH > 5 mU/L but < 9.99 mU/L were retested by a second heel prick and referred if the level remained > 5 mU/L. Referred individuals were subjected to thyroid function tests (TFTs), including T4 and TSH, using a venous blood sample. The time line for performing the confirmatory TFT was chosen according to the TSH level observed in the first screening heel-prick test:

- For TSH < 20 mU/L, around the 21^st^ day.
- For TSH between 20 and 39.9 mU/L, around the 14^th^ day.
- For TSH ≥ 40 mU/L, reassessed and treated on the same day of referral.

**Table 2:** Distribution of all screened newborns and CH patients, based on sex and year of birth

Year of birth^a^	1^st^	2^nd^	3^rd^
All newborns	22 209 (51.2%)^b^	30 021 (50.5%)	34 337 (52%)
CH subjects	39 (48.7%)	85 (48.2%)	70 (55.7%)

a During the study period;

b numbers in parenthesis show the percentage of males.

In the next years of screening, based on our national guidelines, the cutoff for emergency treatment was lowered to ≥ 20 mU/L. The confirmatory test was performed by venipuncture at least 2 hours after the last feeding in the morning. We used an ELISA kit (Monobind, USA) and an ELISA reader (BioTek ELx800, USA) to confirm or rule out CH among the referred suspected neonates. Dealing with the final laboratory results was done according to the protocol of the screening department of the Ministry of Health. This protocol was used with a more definite and detailed algorithm matching international standards so as to avoid unnecessary treatment of transient hyperthyrotropinemia (< 2 months) and to make treatment decisions for infrequent situations (Table 1). All referred suspected infants were followed up on until the final diagnosis was made: the beginning of treatment for CH or until the normalization of thyroid function in transient hyperthyrotropinemia.

The aim was to begin replacement therapy with levothyroxine before the 30^th^ day of life. The CH subjects younger than 6 months were followed up on monthly, between the 6^th^ and 12^th^ month, bimonthly, and after that, until the 36^th^ month, quarterly. The goal was to maintain T4 at > 8 µg/dL, and TSH between 0.5 and 2.0 mU/L, based on the mean normal range (10-12), with normal growth and development. The treatment was stopped earlier than 3 years only if the subjects required < 12.5 µg/day of levothyroxine (1/4 of a 100 µg tablet, every other day) to stay in a euthyroid state. These subjects were classified as transient CH. Subclinical hypothyroidism was defined in the subjects who had normal T4 levels but persistent high TSH (5.5–15 mU/L) after the 60^th^ day (13,14). The patients in whom treatment could be discontinued before 6 months (n = 2) were also classified as transient. However, classification of this group of subjects as transient high TSH would be more appropriate. To compare the final outcome based on the primary diagnostic TSH levels, all cases were classified into three groups: 1) ≥ 40 mU/L; 2) 20-39.9 mU/L; and 3) 5-19.9 mU/L (Table 3).

**Table 3:** Final outcome in 194 cases of CH according to their first serum TSH

TSH*	Permanent; n (%)	F:M	Transient; n (%)	F:M	Unknown; n (%)	F:M	Total; n (%)	F:M
≥ 40	56 (52)†	30:26	39 (36)	19:20	13 (12)	6:7	108 (100)	55:53
20-39.9	10 (25.5)	4:6	25 (64)	11:14	4 (10.5)	2:2	39 (100)	17:22
< 20	15 (32)	6:9	31 (66)	15:16	1 (2)	1:0	47 (100)	22:25
Total	81 (42)	40:41	95 (49)	45:50	18 (9)	9:9	194 (100)	94:100

* mU/L;

† percent of outcome in each TSH group.

**Figure 3:**
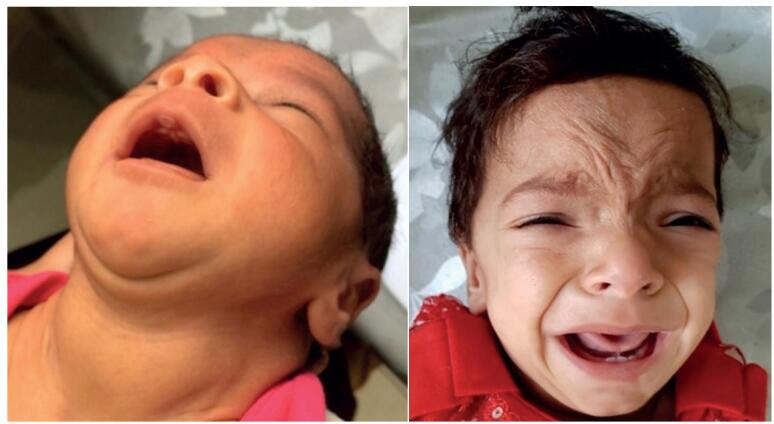
A neonate with goiterous congenital primary hypothyroidism (negative history of maternal thyroid disorder or perinatal risk factor) indicating dyshormonogenesis. She came back after 2 years because of developmental delay. She had a TSH > 100 mU/L due to not taking medication.

After the third birthday, replacement therapy was discontinued for 1 month, then T4 and TSH assessments were performed in all subjects as reevaluation. Any rise of TSH to ≥ 10 mU/L in each test after discontinuation (also in those labeled as transient aged ≤ 3 years) was classified as permanent disease and was followed by thyroid scintigraphy (TS) and re-initiation of levothyroxine therapy. If the T4 and TSH concentrations were in the reference range, euthyroidism was assumed, and a diagnosis of transient hypothyroidism was recorded. On the basis of the TS results, thyroid disorders were classified as agenesis (absence of thyroid on scan), dysgenetic (ectopia, thyroglossal cyst, lingual, hypoplasia, hemi-agenesis, small thyroid, etc.), or dyshormonogenesis (normal scan or diffuse goiter; Figure 3). All those who could leave the treatment before 3 years of age or after the third year (after reevaluation) were followed up on for at least 24 months to distinguish between those with actual transient CH versus permanent subclinical hypothyroidism (TSH ≥ 10 mU/L). Data were presented after 5-year follow-up of all permanent and transient subjects (total 8-year duration of the study) to find the exact prevalence. Because most TSH results were reported in approximation (> 40 or > 100), the TSH ranges, rather than means, were used to compare the outcomes.

### Statistics

Data were analyzed using SPSS version 16.0 (SPSS Inc., Chicago, IL, USA), and the *logistic regression test* was used to analyze risk prediction in each outcome. *P* values < 0.05 were considered statistically significant.

## RESULTS

During the 3 years of screening, 86,567 neonates (51.28% male) were screened, of whom 5,923 (6.8%) had TSH levels ≥ 5 mU/L. Those with TSH ≥ 10 mU/L were referred directly, but a majority of them (n = 5,039; 85%), who had TSH values between 5 and 9.99 mU/L, were reassessed by the heel-prick method and then referred if the second test also revealed TSH to be > 5 mU/L (final recall rate referred on suspicion of CH = 2.4%; this means that up to 10 normal infants were recalled for testing for every 1 case of hypothyroidism). According to the acceptable definitions (Table 1), 194 cases (51.5% male) were diagnosed as CH (both permanent and transient), requiring treatment. The overall incidence was 1:446 live births with a female/male (F/M) ratio of 0.94. Table 2 shows the distribution by year of all live births and the prevalence of CH during the study period.

After the third birthday, all 176 diagnosed CH subjects underwent reevaluation (18 of 194 were missed because of two deaths, five immigrations, and loss of contact information for the rest) with 4 weeks levothyroxine discontinuation and serum T4 and TSH measurement. Based on the TSH (if it was > 10 mU/L in the first or follow-up tests), 81 (46% of 176) subjects were diagnosed with permanent CH (F:M = 40:41; 0.97, similar to the ratio seen in all 194 CH cases, including both permanent and transient). The remaining 95 subjects were followed up on with TSH rechecks for at least 2 more years with intervals of 3, 6, and 12 (and 24 if needed) months to make sure that there was no rise of TSH. These patients were discharged as transient CH. Considering the similar percentage of permanents in the missed group (46%), we probably lost at least 8 (of 18) more permanent CH cases to follow. The final prevalence of permanent CH was calculated as 1:970.

The final outcomes were reviewed and compared in all cases based on their first diagnostic TSH values (Table 3). These data disclosed that 17 of 18 missed cases had TSH > 20 mU/L. This means that we probably lost more than 8 permanent CH cases among the 18 missed cases, indicating a higher prevalence of CH.

Compared to those who had TSH < 20 mU/L (subclinical CH), the patients with TSH ≥ 40 mU/L had at least three times more risk to be permanent (1.41-6.22; 95% CI; OR). For those with TSH = 20-39.9 mU/L, no significant risk was found (0.317-2.15; 95% CI; OR).

TS performed in 53 of 81 permanents showed: agenesis in 5 (F:M = 2:3), dysgenesis in 20 (F:M = 13:7), and normal pattern in 28 (F:M = 11:17), indicating that > 50% of cases were caused by dyshormonogenesis. Details of the TS results are shown in Table 4.

**Table 4:** Comparison of the first serum TSH values in different etiologies of permanent CH based on TS

TS group	TSH ≥ 40*	F:M	20-39.9	F:M	TSH < 20	F:M	Total	F:M
Agenesis	4 (80)†	2:2	1 (20)	0:1	0 (0)	0:0	5 (100)	2:3
Dysgenesis	17 (85)	11:6	1 (5)	1:0	2 (10)	1:1	20 (100)	13:7
Normal	16 (57)	8:8	4 (14.3)	1:3	8 (25)	2:6	28 (100)	11:17
Total	37 (69.8)	21:16	6 (11.3)	2:4	10 (18.8)	3:7	53 (100)	27:26

* mU/L;

† percent of TSH group in each outcome.

Compared to those who had TSH < 20 mU/L (subclinical CH), the patients with TSH ≥ 40 mU/L had almost five times more risk of having agenesis or dysgenesis in TS (0.98-28.2; 95% CI; OR). For those with TSH = 20-39.9 mU/L, no significant risk was found (0.2-19.9; 95% CI; OR).

There were 11 subjects receiving treatment with the diagnosis of mild subclinical CH (TSH = 5.5-10). On reevaluation, four cases were found to be permanent: two boys (both normal scan) and two girls (one dysgenesis and one normal scan). The 7 other subjects were discharged as transient.

During this period, two cases of pan-hypopituitarism (normal ACTH, adrenocorticotropic hormone), two with genetically confirmed Pit-1 defect (deficiency of TSH, growth hormone, and prolactin), and one with isolated TSH deficiency (Figure 2) were also diagnosed independent of the screening program and were included in the overall prevalence without performing TS.

## DISCUSSION

In concurrence with previous reports, we observed a high prevalence of CH in our study, similar to other Asian populations. As per the Iranian Ministry of Health recommendation and our study design, we used TSH as the screening assay to evaluate CH. This probably resulted in missing only 2 of 100 CH cases (12), including those with secondary or tertiary hypothyroidism, infants with an absence of the free T4 feedback mechanism, and those with a delayed elevation of TSH levels. As mentioned above, we found 5 cases of central CH that their missing was unavoidable (Figure 2). Accordingly, the ideal screening method would be to evaluate primary TSH with T4, but it is cost-prohibitive.

As with most congenital disorders, the main causes of the differences in CH prevalence would be ethnicity, gene pool, and the rate of consanguineous marriages (15,16). Similar to other Asian populations, our neonates are at a higher risk for CH, although the main etiologic factors including dysgenesis or dyshormonogenesis were not very prevalent in our cases of CH. In fact, similar to other Iranian studies (17), we found a high rate of transient CH cases (near half), which cannot be attributed to genetics but rather to maternal iodine supplementation and/or thyroid status (7,8). While early treatment of even transient subjects is mandatory to prevent any degree of neurodevelopment impairment, better management of iodine supplementation and maternal thyroid status can obviate a large number of recalls, unnecessary venipuncture, replacement therapy, clinic visits, and, more importantly, family stress and conflict.

After a 2-year follow-up of all those who could leave treatment before or after 3 years of age (including those with mild subclinical CH; TSH < 10 mU/L), we found a higher rate of transients than that previously reported in Isfahan (9) (≈ 50% vs. 40%).

Tc-99m TS was reported to be a useful diagnostic tool for the investigation of suspected CH and can potentially help manage and predict the lifelong replacement therapy requirement (18). However, in the first 3 years of life, the treatment plan remains the same.

The prevalence of permanent CH was found to be about 1/970 live births, which is less than the 1/750 live births reported in Isfahan (9) but near to the 1/918 live births identified in Asian families living in England (19). Using a standard classic approach with a huge number of live births, the prevalence rate of 1/446 (total of permanent and transient cases) found in our study supports the controversial report from central Iran showing a CH incidence of 1/370 (2). The female-to-male ratio for all CH subjects in our study (0.94) was quite similar to that of the whole country (20).

Additionally, many cases of permanent subclinical CH with seemingly normal TS results (except one with a right hemiagenesis) were identified in our study. Because the benefits of replacement therapy are significant even in adults (21), it is logical to treat and follow up on all subclinical CH cases, particularly those with TSH ≥ 10 mU/L. Although patients with TSH levels > 10 mU/L often have reduced free T4 levels and may have hypothyroid symptoms, theoretically, they must be clinically normal regarding the normal T4 levels. The high incidence of permanent subclinical CH in cases with normal T4 but mildly elevated TSH (5–10 mU/L) shows the importance of treatment and follow-up for such infants.

Interestingly, only permanent CH subjects with agenesis/dysgenesis in their scan had a female predominance, similar to what was discovered in CH subjects of Western countries (mostly caused by dysgenesis). The finding of a F:M ≈ 1 in all subjects (permanent and transient cases) and a male predominance in the dyshormonogenesis group is compatible with their etiologic background. Transient CH subjects as a big part of total CH cases possibly caused by environmental factors should not have a sex difference. Dyshormonogenesis is almost always inherited in a recessive pattern, but only 2% of dysgenesis is familial (22). This may explain the F:M differences we discovered in two groups of thyroid dyshormonogenesis and thyroid dysgenesis.

In concordance with previous studies (17,23), in our study, patients with permanent CH had higher TSH levels than transient ones during the neonatal period. This finding emphasizes the need for careful follow-up in those with TSH ≥ 40 mU/L.

The limitations of this study include inexperienced laboratory technicians, health staff, and physicians, particularly in the first year of the study (24,25); non-cooperative hospitals; families who did not participate or who approached private clinics independently; and the inability to achieve 100% screening coverage. Table 2 displays the number of screened newborns, rather than live births, which may have resulted in underestimating the total number of CH cases, although this problem theoretically does not change the prevalence recorded by our study.

The strength of this study is that it determined the real percentage of transient CH cases and also identified those cases with permanent CH but normal TS with a high degree of accuracy.

In conclusion, the prevalence of CH in the southwest part of Iran was found to be much higher than that of the Western countries; however, it was less than that found in central Iran and similar to that seen in Asian families living in Western countries. Accordingly, due to the importance of replacement therapy for transient cases, adequate follow-up is necessary. The high incidence of transient cases suggests environmental and/or maternal causes, rather than a genetic basis for the CH.
